# Unilateral Optic Nerve Sheath Fenestration in Idiopathic Intracranial Hypertension: A 6-Month Follow-Up Study on Visual Outcome and Prognostic Markers

**DOI:** 10.3390/life11080778

**Published:** 2021-07-31

**Authors:** Snorre Malm Hagen, Marianne Wegener, Peter Bjerre Toft, Kåre Fugleholm, Rigmor Højland Jensen, Steffen Hamann

**Affiliations:** 1 Department of Ophthalmology, Rigshospitalet, University of Copenhagen, 2600 Glostrup, Denmark; marianne.wegener.01@regionh.dk (M.W.); peter.bjerre.toft@regionh.dk (P.B.T.); steffen.ellitsgaard.hamann@regionh.dk (S.H.); 2 Department of Neurosurgery, Rigshospitalet, University of Copenhagen, 2100 Copenhagen Ø, Denmark; kaare.fugleholm.buch@regionh.dk; 3 Danish Headache Center, Department of Neurology, Rigshospitalet-Glostrup, University of Copenhagen, 2600 Glostrup, Denmark; rigmor.jensen@regionh.dk

**Keywords:** optic nerve sheath fenestration, idiopathic intracranial hypertension, papilledema, optic nerve head, automated perimetry, optical coherence tomography

## Abstract

Loss of vision is a feared consequence of idiopathic intracranial hypertension (IIH). Optic nerve sheath fenestration (ONSF) may be an effective surgical approach to protect visual function in medically refractory IIH. In this study, we evaluate the impact of unilateral superomedial transconjunctival ONSF on bilateral visual outcome using a comprehensive follow-up program. A retrospective chart review of IIH patients who underwent unilateral ONSF between January 2016 and March 2021 was conducted. Patients fulfilling the revised Friedman criteria for IIH and who had exclusively received ONSF as a surgical treatment were included. Main outcomes were visual acuity (VA); perimetric mean deviation (PMD); papilledema grade; and optic nerve head elevation (maxONHE) 1 week, 2 weeks, and 1, 3, and 6 months after surgery. VA (*p* < 0.05), PMD (*p* < 0.05), papilledema grade (*p* < 0.01), and maxOHNE (*p* < 0.001) were improved after 6 months on both the operated and non-operated eye. Prolonged surgical delay impedes PMD improvement (r = −0.78, *p* < 0.01), and an increasing opening pressure initiates a greater ganglion cell loss (r = −0.79, *p* < 0.01). In this small case series, we demonstrate that unilateral superonasal transconjunctival ONSF is a safe procedure with an effect on both eyes. Optic nerve head elevation and PMD are feasible biomarkers for assessing early treatment efficacy after ONSF.

## 1. Introduction

Idiopathic intracranial hypertension (IIH) is a condition characterized by raised intracranial pressure (ICP) of unknown etiology, mainly affecting young and obese females [1]. The most common symptoms are debilitating chronic headache, pulsatile tinnitus, and visual disturbances. In most cases, visual acuity is normal and visual fields show localized nerve fiber bundle defects with or without enlarged blind spots [2,3]. If IIH is not managed sufficiently with a marked reduction in ICP there is an imminent risk of permanent visual impairment due to secondary optic nerve atrophy. Peroral medication with the carbon anhydrase inhibitor acetazolamide (Diamox^®^), or the less rigorously evaluted topiramate (Topimax^®^), and weight loss are effective long term treatments in mild to moderate cases of IIH [4,5]. In severe cases with an urgent need for more acute interventions, surgical treatment is a cornerstone in treatment.

Surgical treatment for IIH is recommended in medically refractory cases, non-compliance, or in cases with severe and rapid decline in visual function. Neurosurgical cerebrospinal fluid (CSF) diversion is generally considered effective for immediate stabilization of the condition, and shunt treatment is the most common surgical approach in IIH [6,7]. Shunt treatment, however, is susceptible to high failure rates and multiple surgical revisions [8]. An emerging treatment for IIH is venous sinus stenting (VSS), which is an invasive neuro-radiological procedure reducing a trans-stenotic pressure gradient in the venous sinuses and thereby lowering ICP. Optic nerve sheath fenestration (ONSF) is reported to be an effective surgical treatment option with few complications [9]. Reduction of the elevated pressure in the subarachnoid space is achieved by surgical fenestration of the perioptic dura and arachnoid membranes immediately behind the globe. Several surgical techniques for accessing the optic nerve sheath in the orbital cavity have been previously described [10,11,12,13]. A medial transconjunctival, a superomedial transconjunctival, a superomedial transcutaneous upper eyelid, or a lateral orbitomy approach are preferred among performing surgeons [14]. Although no prospective trials have yet evaluated the surgical approaches for optic nerve decompression with ONSF, the overall complication and failure rate is better compared to shunt treatment and equal to VSS [9]. Unilateral ONSF has previously been effective with early improvement on papilledema reduction on the contralateral non-operated eye [15]. Optical coherence tomography (OCT) and automated perimetry are important tools for IIH management [16,17]. To our knowledge, the value of visual field testing and OCT of the optic nerve head (ONH) remains to be elucidated in the early post-operative stage of unilateral ONSF.

The present study assesses the safety and efficacy of a superonasal transconjunctival surgical approach for unilateral ONSF, with special emphasis on preoperative characteristics and postoperative visual outcome as well as structural optic nerve head changes in a 6-month continuous follow-up perspective.

## 2. Materials and Methods

### 2.1. Data Collection

A retrospective chart review of all patients with an IIH diagnosis (G93.2) who underwent unilateral ONSF in a single tertiary hospital (Rigshospitalet, Capital Region, Denmark) between January 2016 and March 2021 was conducted. For inclusion, the patients had to fulfill the revised Friedman criteria for IIH [1]. Patients were excluded if other surgical or invasive techniques were applied within 6 months of ONSF (e.g., shunt placement or VSS).

The data collection included gender, age, body mass index (BMI), lumbar puncture opening pressure (LOP), time from diagnosis to surgery, pre-operative optic nerve sheath diameter (ONSD measured 3 mm behind the globe on orbital magnetic resonance imaging (MRI) axial T2 imaging sequences), medication, and post-surgical adverse events. Main outcomes were best corrected visual acuity (BCVA, in logMAR converted from Snellen), visual field (VF) testing with perimetric mean deviation (PMD, in dB), papilledema grade, OCT with measurement of maximum optic nerve head elevation (maxONHE, in µm), and macular ganglion cell volume (GCLvol, in mm^3^). The postoperative follow-up time points were 1 week (3–9 days), 2 weeks (10–21 days), 1 month (22–59 days), 3 months (60–134 days), and 6 months (135- days). Optic disc photos were graded by two blinded neuro-ophthalmologists (S.H. and M.W.) using the modified Frisén grading scale [18,19]. VF testing was performed with a 30-2 pattern dynamic program (Octopus 900, Haag-Streit Diagnostics). OCT scans were performed on a system with a built-in follow-up program (SPECTRALIS^®^ OCT, Heidelberg Engineering Inc., Heidelburg, Germany).

### 2.2. Non-Surgical Treatment

From the time of diagnosis, all patients received a total daily dose of up to 3000 mg of acetazolamide (Diamox^®^). In patients with contraindications (n = 2) or prominent side effects (n = 2) a lower daily dose (ranging from 0 mg to 2000 mg) of acetazolamide were used. Medical treatment was continued postoperatively and gradually withdrawn after 6 months. All patients were offered a clinical dietary consultation for weight loss.

### 2.3. Surgical Procedure

Indication for ONSF was in all cases based on the local clinical guideline (IIH-diagnosis + papilledema evaluated by neuroophthalmologist + increased ICP (>25 cm CSF) + distended optic nerve sheath on MRI + insufficient efficacy of medical treatment). The surgical technique used was a superonasal transconjunctival orbitomy, which is a modified version of the medial transconjunctival orbitomy [20]. All ONSFs in this study were performed by an oculoplastic surgeon (P.B.T.) and a neurosurgeon (K.F.) in collaboration. Using a surgical microscope, the conjunctiva was opened with scissors in the superonasal quadrant 3 mm from and parallel to the limbus. The Tenon’s capsule under the incision was excised. The medial rectus and the inferior rectus were secured with sutures and the eye was rotated in the infero-temporal direction. The optic nerve was identified by using malleable retractors and manipulation of the orbital fat with a curved elevator and the suction tip. Then, an approx. 3 × 2 mm^2^ window was cut in the optic nerve sheet with scissors, which resulted in CSF appearing in the field. A dissector was passed in the subarachnoid space to release any adhesions. The conjunctiva was closed with a 7-0 suture (Vicryl Rapide (polyglactin 910), Ethicon, Inc., Raritan, NJ, USA). A video (by P.B.T. and K.F.) is available on online [21].

### 2.4. Statistical Analisys

Data are presented as mean ± standard deviation if not specified otherwise. Repeated measures with a mixed-effects model and Dunnett’s multiple comparisons post hoc test were used for continuous data, calculating mean differences from baseline to every follow-up time point. Simple linear regression analyses were performed, and correlations were computed according to normality distribution of the data. The Shapiro–Wilk test was used for the normality test. All statistical analyses were performed in GraphPad Prism version 9. *p*-values were calculated as two-tailed and considered statistically significant if below 0.05.

## 3. Results

### 3.1. Demographics

One hundred seventy-one patients were identified with an IIH-diagnosis in our tertiary multidisciplinary specialized IIH-center in the study period. Ten patients fulfilled the inclusion criteria with unilateral ONSF. All were female with a mean age of 28.7 ± 11.4 years at time of surgery. The baseline body mass index (BMI) was 34.4 ± 4.8 kg/m^2^ with a mean decrease of 2.2 kg/m^2^ (95% CI {−4.5, 0.1}, *p* = 0.06) after 6 months. All patients fulfilled the revised Friedman criteria for IIH with bilateral papilledema (grade 2 to 5) and a mean LOP of 50.3 ± 12.0 cm CSF. All preoperative demographics are listed in Table 1. In all cases, the ONSF was performed unilaterally on the worst eye based on VF test results. In one case, a second fenestration was performed on the fellow eye 1 month later due to insufficient treatment effect on that eye (data after the secondary ONSF are excluded from continuous analysis). The median time from diagnosis to primary fenestration was 9.5 days (range [5, 76]). Minor postoperative complications in the form of transient sub-conjunctival bleeding or inflammation (n = 5), chemosis and periorbital edema (n = 2), and lacrimation (n = 1) were registered. No postoperative infections or severe complications were seen in any of the cases.

### 3.2. Visual Acuity

**Figure 1:**
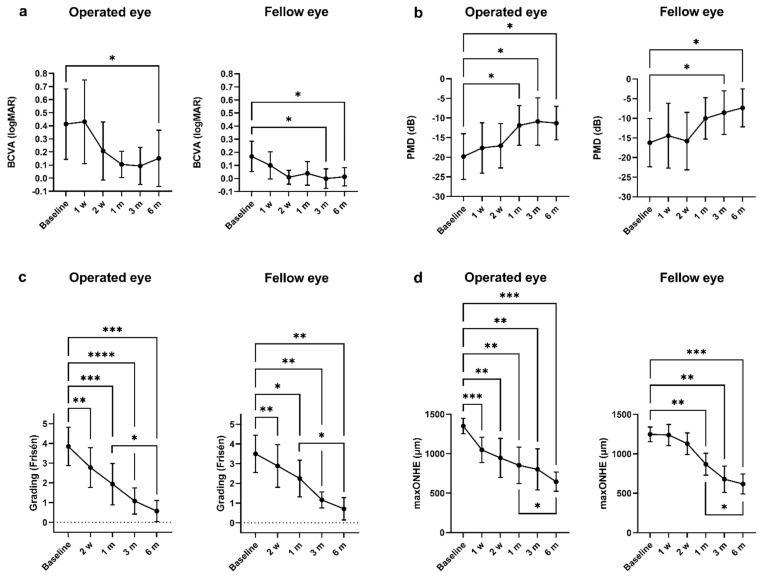
Optic nerve sheath fenestration and primary outcomes: Continuous data from baseline to 6 months. (**a**) Mean best corrected visual acuity (BCVA) for the operated and fellow eye improved 6 months postoperatively (*p* < 0.05). However, an improvement could already be observed after 1 month. (**b**) Perimetric mean deviation (PMD) improved after 1 month in the operated eye (*p* < 0.05) and after 6 months in the fellow eye (*p* < 0.05). There was no further improvement in PMD from 1 month to 6 months in any of the eyes (*p* > 0.5). (**c**) The mean modified Frisén papilledema grade improved in both eyes after 2 weeks (*p* < 0.05) and continued to improve until the 6-month follow-up, with a statistically significant change from 1 month to 6 months. (**d**) The maximum optic nerve head elevation (maxONHE) obtained from optical coherence tomography was reduced 1 week postoperatively (*p* < 0.001). In the fellow eye, a statistically significant reduction was reached after 1 month. A further reduction was observed from the 1-month to the 6-month follow-up (*p* < 0.05). (**a**–**d**) Repeated measures mixed-effects model followed by Dunnett’s Test. Data are presented as mean with 95% confidence interval. * = *p* < 0.05, ** = *p* < 0.01, *** = *p* < 0.001, **** = *p* < 0.0001.

Baseline BCVA was logMAR 0.41 ± 0.38 on the operated eye and 0.17 ± 0.16 on the fellow eye with no statistically significant difference (*p* > 0.05). BCVA on the operated eye improved in 3 (30%) and remained stable in 7 (70%) 1 month postoperatively, with no changes at the 6-month follow-up. In the fellow eye, BCVA improved in 1 (10%) and stabilized in 9 (90%) after 1 month from surgery and improved in 3 (30%) and stabilized in 7 (70%) after 6 months from surgery. After 6 months from surgery, BCVA on the operated eye improved by −0.26 (95% CI [−0.48, −0.05]) from baseline (*p* = 0.04), and the fellow eye improved by −0.16 (95% CI [−0.30, −0.01]) from basline (*p* = 0.04) (Table 2, Figure 1a). After a 6 month follow-up, no statistically significant difference in BCVA was found between the operated and the fellow eye. Individual data as well as linear regression can be found in Supplementary Figure S1.

### 3.3. Visual Fields

Visual fields at baseline showed that PMD was −19.8 ± 7.6 dB on the operated eye and −16.2 ± 8.0 dB on the fellow eye, with no statistically significant difference (*p* > 0.05). After 1 month, the operated eye improved by 7.9 dB (95% CI [1.4, 14,4] from baseline (*p* = 0.02) and kept improving until the 6-month follow-up with a final value of −11.3 ± 4.6 dB (*p* = 0.02) (Table 2, Figure 1b). In comparison, the fellow eye showed a slower improvement in PMD but with a statistically significant improvement compared to baseline after 6 months with a final value of −7.3 ± 5.2 dB (*p* = 0.03) (Table 2, Figure 1b). No statistically significant differences in PMD were found between the operated and the fellow eye at any of the follow-up time points. Individual data as well as linear regression can be found in Supplementary Figure S1.

### 3.4. Papilledema Grading and Maximum Optic Nerve Head Elevation (maxONHE)

The mean baseline papilledema grade for the operated and fellow eye were 3.9 ± 1.0 and 3.5 ± 0.9, respectively. A decrease in papilledema grade was seen in both eyes after 2 weeks, with a continuous decrease in grade until 6 months (*p* < 0.05) (Table 2, Figure 1c). The baseline maxONHE on the operated eye was 1351 ± 128 µm and 1248 ± 120 µm on the fellow eye, with no significant difference (*p* > 0.05). On the operated eye, a postoperative effect on papilledema reduction was detectable after 1 week compared to baseline (*p* = 0.0003), and the improvement continued until the 6-month follow-up (*p* = 0.0003) (Table 2, Figure 1d). A delayed surgical response was observed on the fellow eye where a significant postoperative papilledema reduction was reached after 1 month (*p* = 0.007) (Figure 1d). No statistically significant differences were found between maxONHE in the operated and fellow eye at any of the follow-up time points. Individual data as well as linear regression can be found in Supplementary Figure S2.

### 3.5. Preoperative Prognostic Factors and Short-Term Postoperative Improvements on Long-Term (6 Month) Outcome

Baseline median GCL volume for the operated and non-operated eye were within the normal range [22] with 1.07 mm^3^ (range [0.46, 1.25]) and 1.09 mm^3^ (range [0.73, 1.36]), respectively. At 6-month follow-up, the operated eye had a median GCL volume loss of −0.23 mm^3^ (95% CI [−0.27, −0.00]) and the fellow eye had a mean loss of −0.13 mm^3^ (95% CI [−0.34, −0.06]) from baseline (Table 2).

Days from diagnosis to surgery correlated negatively with PMD improvement (Spearman, r = −0.78, *p* = 0.0016). An invert correlation was found between the baseline LOP and the GCL volume change from baseline (Spearman, r = −0.79, *p* = 0.0011), indicating that an increasing opening pressure initiates a greater ganglion cell loss. No correlation was found between the baseline LOP and improvement in BCVA or PMD. However, a moderate inverse correlation was observed between baseline LOP and the final PMD value (Spearman, r = −0.52, *p* = 0.04). All linear regression scatter plots are located in Supplementary Figure S3.

Regarding BCVA and VF, there was no further statistically significant improvement in visual function after 1 month (*p* > 0.5) (Figure 1a,b). Conversely, papilledema grade and elevation, reflecting the structural changes of the ONH, did continue to decrease until 6 months (*p* < 0.05) (Figure 1c,d).

## 4. Discussion

ONSF is a surgical procedure performed to decompress the optic nerves and alleviate vision threatening papilledema in the setting of raised ICP. In this small single-center case series, we found the superonasal transconjunctival approach for unilateral ONSF safe and effective on rapid papilledema reduction on both the operated and non-operated eye in patients with vision threatening IIH. This was also reflected functionally in VF testing with a significant improvement in PMD after 1 month and significant improvement in VA after 6 months.

The superonasal transconjunctival surgical technique is a modified version of the medial transconjunctival orbitomy [20]. In contrast to the medial approach, the superonasal transconjunctival approach respects the insertion of the medial rectus muscle, thus the risk of postoperative diplopia is reduced. We observed no postoperative diplopia or other serious or permanent adverse events in any of the patients, using this technique for ONSF. Recently, in the first study of its kind, bilateral superonasal transconjunctival ONSF in 66 IIH patients was found to be safe and effective after 6 weeks [23]. Similar to our results, the papilledema grade and VF improved significantly within 6 weeks, with no significant change in VA.

Although this current study does not explain the mechanisms behind ONSF, the contralateral effect on papilledema reduction and visual function could potentially be based on the previously proposed CSF filtration effect with a general ICP decrease [24,25]. The general ICP decrease is supported by improvement in headache symptoms, where a large systematic review found overall headache resolution in 49.3% of patients after ONSF versus 69.8% in CSF diversion [9]. Because bilateral ONSF appears to favor better headache outcome than unilateral operation [26] we suppose that the degree of CSF filtration equals the decrease in ICP, and even a slight decrease can stabilize or improve visual function. However, this postulation has to be proven by continuous pre- and postoperative ICP monitoring using ICP waveform analysis.

### 4.1. Visual Function

Visual field testing with automated perimetry is an important tool for monitoring visual impairment in IIH with papilledema. We found a significant postoperative improvement in VF (PMD) 1 month after surgery in the operated eye and after 6 months in the fellow eye. ONSF has earlier shown a significant effect on VF improvement, but to a lesser extent than our findings, which could be explained by a worse preoperative PMD in our patients [10,11,23]. Interestingly, we observed that the final mean PMD in the non-operated eye was better than in the operated eye. We suggest that this observation is due to the less negative preoperative PMD value in the non-operated eye. In general, an abnormal PMD is interpreted as a functional measurement of neuroretinal damage (ganglion cell death and/or dysfunction). We found a general decline in macular ganglion cell volume in both eyes post-operatively. We suggest that this reflects a permanent structural damage occurring before any treatment was initiated.

We found a marked improvement in VA in both eyes after 6 months but failed to show any improvement in the early postoperative period (1 week to 1 month). However, no VA deterioration was seen, VA being either stabilized (70%) or improved (30%) after 1 month. Our VA findings in this study are in line with previous studies [11,23,27].

Overall, in this small case series we found VA to be less valuable for tracking visual improvement in the early postoperative phase and that it is inferior to VF.

### 4.2. Papilledema

Unilateral ONSF has previously shown significant and bilateral reduction of papilledema after 2 weeks, with further reduction after 3, 6, and 12 months using Frisén grading [15]. In our study, we found the same effect on papilledema reduction. Further, we have demonstrated that changes in the ONH morphology after unilateral ONSF can be detected in both eyes within weeks after surgery using the built-in follow-up scanning protocol provided by the Heidelberg OCT system. Measuring the height of the ONH on OCT has earlier been described as a feasible method for tracking papilledema changes [28]. This method was refined using the ONH volume, demonstrating a strong correlation to the Frisén grade, and also serving as a surrogate for ICP changes [29,30]. In the current study, we used a modified version, measuring maxONHE for tracking papilledema reduction.

### 4.3. Limitations and Strengths

The main limitation of this study is the small number of patients, making it unsuitable for drawing clear conclusions and performing stratification and multivariate analysis. Additionally, parallel drug treatment and weight loss could have influenced the ONSF treatment effect in a positive way. However, we did not see a significant change in BMI during the 6-month follow-up. This study was unsuitable for evaluating the impact of the medical therapy, and this should be addressed in future studies. Missing data were due to the relocation of patients (n = 1) and loss to follow-up (n = 1). A strength in this single-center study is the meticulous diagnostic workup and follow-up program with single-system automated perimetry and OCT-scanning protocol. Further it emphasized the need for a dedicated multidisciplinary team for IIH patients and a careful selection of patients for invasive procedures.

### 4.4. Correlations and Perspectives

Early surgical treatment in selected cases favors better VF outcome in IIH [31]. Equally, we found a significant invert correlation between days from diagnosis to surgery and improvement in PMD, which addresses the importance of an efficient diagnostic workup and advances the decision on surgery.

Interestingly, we found that a high preoperative LOP caused a greater GCL volume loss despite surgical and medical therapy. Additionally, a moderate inverse correlation between LOP and final PMD after 6 months was observed. We suggest that raised ICP, and in this case high LOP, may serve as a viable prognostic marker for permeant neuroretinal damage and a worse VF outcome. Therefore, medically treated IIH patients identified with very high LOP might benefit from ICP monitoring with either repeated LOP or a continuous telemetric ICP measurement. Regardless of permanent neuroretinal degeneration, we found ONSF effective on improving visual function and papilledema after 6 months. This indicates that ONSF is a good surgical treatment for patients with a high preoperative LOP (≥50 cm CSF), which previously has been questioned [32].

In summary, a unilateral superonasal transconjunctival ONSF approach is a safe and effective treatment for acute IIH patients with compromised vision. Optic nerve head elevation and visual field testing with automated perimetry are both viable biomarkers for assessing early treatment efficacy after ONSF and can thereby determine if additional surgical treatment is required. High preoperative LOP and delayed surgical treatment appears to have a negative impact on long-term neuroretinal morphology and visual function, respectively.

## Figures and Tables

**Table 1:** Preoperative demographics.

Patients, n	10			
Gender, n (%)	Female, 10 (100)			
			Range	
Age, mean	28.7 ± 11.4 years		[16.4, 46.3]	
Body mass index, mean ± SD	34.4 ± 4.8 kg/m^2^		[29.1, 42.3]	
Opening pressure, mean ± SD	50.3 ± 12.0 cm CSF		[31, 651]	
Diagnosis to surgery, median	10 days		[5, 76]	
	Operated eye	Range	Fellow eye	Range
ONSD, median	7 mm	[5, 8]	7 mm	[5, 7]

SD = Standard deviation. CSF = Cerebrospinal fluid. ONSD = Optic nerve sheath diameter measured 3 mm behind the globe on an axial T2-weighted magnetic resonance image of the orbits. Opening pressures stated as >50 cm CSF (n = 3) in the patient chart are adjusted to 65 cm CSF, which approximates to the excess tubing of the standard manometer.

**Table 2:** Optic nerve sheath fenestration and primary outcomes: Baseline values and 1- and 6-months changes.

	Baseline		1 Month		6 Months		1 Month–Baseline Diff.		6 Months–Baseline Diff.	
Number of Patient, n	10		8		8					
	Operated eye	Fellow eye	Operated eye	Fellow eye	Operated eye	Fellow eye	Operated eye	Fellow eye	Operated eye	Fellow eye
BCVA (logMAR)										
Mean ± SD	0.41 ± 0.38	0.17 ± 0.16	0.11 ± 0.12	0.04 ± 0.11	0.15 ± 0.23	0.01 ± 0.06	−0.31	−0.13	−0.26 *	−0.16 *
Range [min, max]	[−0.08, 1.30]	[0.00, 0.52]	[−0.08, 0.30]	[−0.08, 0.22]	[−0.08, 0.62]	[−0.08, 0.10]				
95% CI diff.							[−0.74, 0.12]	[−0.32, 0.06]	[−0.48, 0.05]	[−0.30, −0.01]
Snellen equivalent	0	1	1	1	1	1				
PMD (dB)										
Mean ± SD	−19.8 ± 7.6	−16.2 ± 8.0	−11.9 ± 6.0	−10.0 ± 6.3	−11.3 ± 4.6	−7.3 ± 5.2	7.9 *	6.2	8.5 *	8.9 *
Range [min, max]	[−27.8, −7.3]	[−25.9, −2.3]	[−19.0, −2.8]	[−18.7, −0.2]	[−18.7, −6.4]	[−16.7, −2.6]				
95% CI diff.							[1.4, 14.4]	[1.7, 14.1]	[1.8, 15.2]	[1.3, 16.4]
Papilledema grade										
Mean ± SD	3.9 ± 1.0	3.5 ± 0.9	1.9 ± 1.1	2.3 ± 0.9	0.6 ± 0.5	0.7 ± 0.6	−1.9 ***	−1.3 *	−3.3 ***	−2.8 **
Range [min, max]	[2, 5]	[2, 5]	[1, 4]	[1, 4]	[0, 1.5]	[0, 1.5]				
95% CI diff.							[−2.7, −1.2]	[−2.3, −0.2]	[−4.3, −2.3]	[−4.2, −1.3]
maxONHE (µm)										
Mean ± SD	1351 ± 128	1248 ± 120	853 ± 276	868 ± 165	646 ± 116	618 ± 136	−498 **	−380 **	−705 ***	−630 ***
Range [min, max]	[1130, 1565]	[980, 1353]	[538, 1315]	[598, 1133]	[496, 809]	[451, 841]				
95% CI diff.							[−775, −222]	[−635, −126]	[−927, −484]	[−889, −371]
Macular GCLvol (mm^3)^										
Mean ± SD	1.07	1.09	0.95	1.09	0.9	1.08	−0.09 *†	−0.08 **†	−0.23 *†	−0.13 †
Range [min, max]	[0.46, 1.25]	[0.73, 1.36]	[0.46, 1.10]	[0.69, 1.26]	[0.79, 1.00]	[0.82, 1.14]				
95% CI diff.							[−0.17, 0.00]	[−0.11, −0.01]	[−0.34, −0.06]	[−0.27, 0.00]

BCVA = Best corrected visual acuity. PMD = Perimetric mean deviation. maxONHE = Maximum optic nerve head elevation measured with optical coherence tomography (OCT) B-scan. GCLvol = Ganglion cell layer volume measured on OCT raster B-scan of the macula. CI diff. = Confidence interval for the difference. † Wilcoxon matched-pairs signed rank test. * = *p* < 0.05, ** = *p* < 0.01, *** = *p* < 0.001.

## Supplementary Materials

The following are available online at https://www.mdpi.com/article/10.3390/life11080778/s1, Figure S1: Supplemental data 1: Visual function, Figure S2: Supplemental data 2: Optic nerve head structure, Figure S3: Supplemental data 3: Correlations.
