# Erratum: Hagen et al. Unilateral Optic Nerve Sheath Fenestration in Idiopathic Intracranial Hypertension: A 6-Month Follow-Up Study on Visual Outcome and Prognostic Markers. *Life* 2021, *11*, 778

**DOI:** 10.3390/life11101014

**Published:** 2021-09-27

**Authors:** Snorre Malm Hagen, Marianne Wegener, Peter Bjerre Toft, Kåre Fugleholm, Rigmor Højland Jensen, Steffen Hamann

**Affiliations:** 1 Department of Ophthalmology, Rigshospitalet, University of Copenhagen, 2600 Glostrup, Denmark; marianne.wegener.01@regionh.dk (M.W.); peter.bjerre.toft@regionh.dk (P.B.T.); steffen.ellitsgaard.hamann@regionh.dk (S.H.); 2 Department of Neurosurgery, Rigshospitalet, University of Copenhagen, 2100 Copenhagen Ø, Denmark; kaare.fugleholm.buch@regionh.dk; 3 Danish Headache Center, Department of Neurology, Rigshospitalet-Glostrup, University of Copenhagen, 2600 Glostrup, Denmark; rigmor.jensen@regionh.dk

It has come to our attention that there has been an error in the previous work [1]. Due to an error during copy editing, some of the “Snellen equivalent” data in the Table 2 are incorrect in the published paper.

The data in Table 2 (Line Snellen equivalent) were corrected from “0/1/1/1/1/1” to “0.4/0.6/0.8/1.0/0.6/1.0”. This change does not affect the conclusion of the experiment.

**Table 2:** Optic nerve sheath fenestration and primary outcomes: baseline values and 1- and 6-months changes.

	Baseline		1 Month		6 Months		1 Month—Baseline Diff.		6 Months—Baseline Diff.	
Number of Patient, n	10		8		8					
	Operated Eye	Fellow Eye	Operated Eye	Fellow Eye	Operated Eye	Fellow Eye	Operated Eye	Fellow Eye	Operated Eye	Fellow Eye
BCVA (logMAR)										
Mean ± SD	0.41 ± 0.38	0.17 ± 0.16	0.11 ± 0.12	0.04 ± 0.11	0.15 ± 0.23	0.01 ± 0.06	−0.31	−0.13	−0.26 *	−0.16 *
Range [min, max]	[−0.08, 1.30]	[0.00, 0.52]	[−0.08, 0.30]	[−0.08, 0.22]	[−0.08, 0.62]	[−0.08, 0.10]				
95% CI diff.							[−0.74, 0.12]	[−0.32, 0.06]	[−0.48, 0.05]	[−0.30, −0.01]
Snellen equivalent	0.4	0.6	0.8	1.0	0.6	1.0				
PMD (dB)										
Mean ± SD	−19.8 ± 7.6	−16.2 ± 8.0	−11.9 ± 6.0	−10.0 ± 6.3	−11.3 ± 4.6	−7.3 ± 5.2	7.9 *	6.2	8.5 *	8.9 *
Range [min, max]	[−27.8, −7.3]	[−25.9, −2.3]	[−19.0, −2.8]	[−18.7, −0.2]	[−18.7, −6.4]	[−16.7, −2.6]				
95% CI diff.							[1.4, 14.4]	[1.7, 14.1]	[1.8, 15.2]	[1.3, 16.4]
Papilledema grade										
Mean ± SD	3.9 ± 1.0	3.5 ± 0.9	1.9 ± 1.1	2.3 ± 0.9	0.6 ± 0.5	0.7 ± 0.6	−1.9 ***	−1.3 *	−3.3 ***	−2.8 **
Range [min, max]	[2, 5]	[2, 5]	[1, 4]	[1, 4]	[0, 1.5]	[0, 1.5]				
95% CI diff.							[−2.7, −1.2]	[−2.3, −0.2]	[−4.3, −2.3]	[−4.2, −1.3]
maxONHE (µm)										
Mean ± SD	1351 ± 128	1248 ± 120	853 ± 276	868 ± 165	646 ± 116	618 ± 136	−498 **	−380 **	−705 ***	−630 ***
Range [min, max]	[1130, 1565]	[980, 1353]	[538, 1315]	[598, 1133]	[496, 809]	[451, 841]				
95% CI diff.							[−775, −222]	[−635, −126]	[−927, −484]	[−889, −371]
Macular GCLvol (mm^3)^										
Mean ± SD	1.07	1.09	0.95	1.09	0.9	1.08	−0.09 *†	−0.08 **†	−0.23 *†	−0.13 †
Range [min, max]	[0.46, 1.25]	[0.73, 1.36]	[0.46, 1.10]	[0.69, 1.26]	[0.79, 1.00]	[0.82, 1.14]				
95% CI diff.							[−0.17, 0.00]	[−0.11, −0.01]	[−0.34, −0.06]	[−0.27, 0.00]

BCVA = Best corrected visual acuity. PMD = Perimetric mean deviation. maxONHE = Maximum optic nerve head elevation measured with optical coherence tomography (OCT) B-scan. GCLvol = Ganglion cell layer volume measured on OCT raster B-scan of the macula. CI diff. = Confidence interval for the difference. † Wilcoxon matched-pairs signed rank test. * = *p* < 0.05, ** = *p* < 0.01, *** = *p* < 0.001.
