# Supplementary figures and images for: Unilateral Optic Nerve Sheath Fenestration in Idiopathic Intracranial Hypertension: A 6-Month Follow-Up Study on Visual Outcome and Prognostic Markers
Source: Life (Basel). 2021 Jul 31;11(8):778. doi: 10.3390/life11080778 (PMC8400184; doi:10.3390/life11080778)

# Supplemental data 3: Correlations

- Operated eye
- Fellow eye

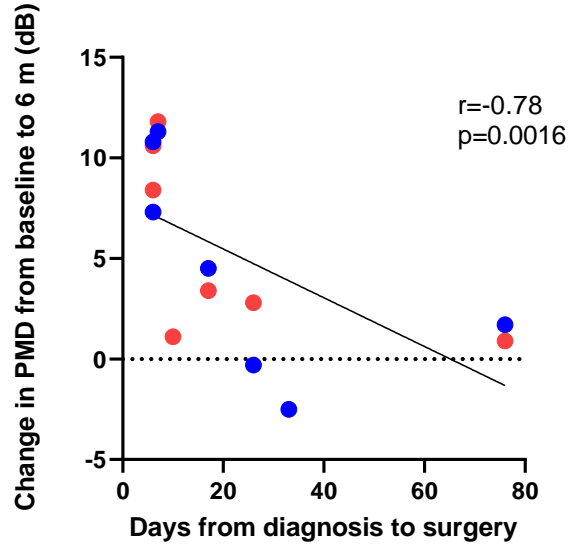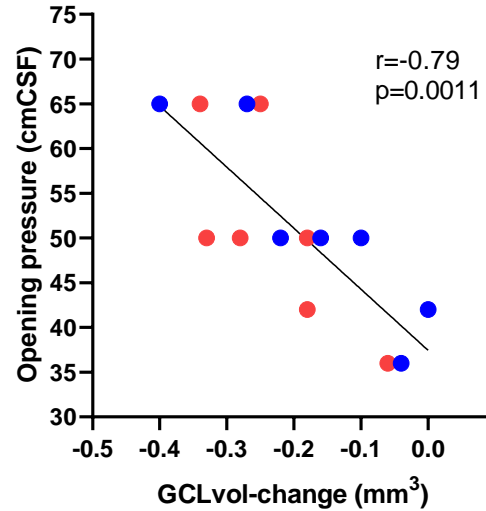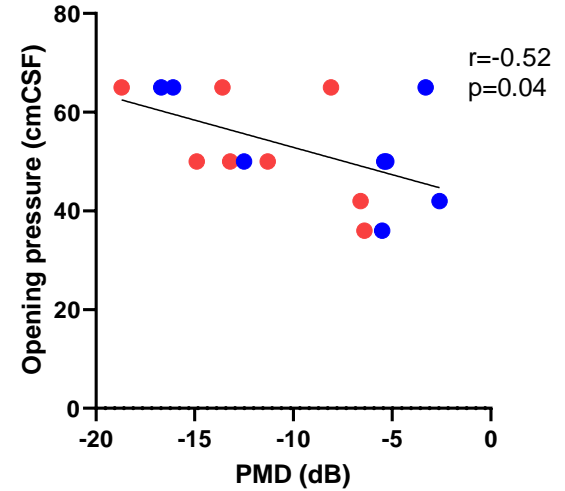

Supplement: Supplementary file 1 [file life-11-00778-s001.zip › Figure S3.pdf]
